# Sorting nexin 10 knockdown: new strategies for alleviating sepsis-associated acute lung injury

**DOI:** 10.1590/1414-431X2025e15117

**Published:** 2026-02-16

**Authors:** Min Wei, Jian Yang, Zejia Yu, Shan Li, Hui Xie, Shiyang Yuan, Qiujie Huang, Hui Zhang, Jun Feng

**Affiliations:** 1 Department of Critical Care Medicine, Fujian Medical University Union Hospital, Fuzhou, China; 2 Department of Critical Care Medicine, Nanping First Hospital Affiliated to Fujian Medical University, Nanping, China

**Keywords:** Acute lung injury, Sorting nexin 10, Oxidative stress, NF-κB/NLRP3 pathway, Inflammation

## Abstract

Acute lung injury (ALI) is a common complication of sepsis in connection with excessive inflammation and accumulation of oxidative stress. Sorting nexin 10 (SNX10) is a sorting nexin family member involved in inflammatory processes. This study aimed to explore the function of SNX10 in ALI. The cecal ligation and puncture (CLP) model was established to induce ALI in C57BL/6J mice. CLP mice exhibited elevated levels of SNX10 expression in the lung tissues. Mice were intratracheally injected with 50 μL adenovirus (10^8^ PFU) containing short hairpin RNA plasmid targeting SNX10. SNX10 knockdown mice showed remission of CLP-induced pulmonary edema, hemorrhage, inflammatory infiltration, and thickened alveolar septum. SNX10 downregulation reduced reactive oxygen species (ROS) levels, increased superoxide dismutase activity and glutathione content, and decreased malondialdehyde content in the lung tissues. SNX10 knockdown decreased the phosphorylation of NF-κB p65 and its nuclear translocation, thus inhibiting the levels of tumor necrosis factor (TNF)-α and interleukin (IL)-6. Furthermore, SNX10 downregulation inhibited the NLRP3, p20 caspase 1, and ASC protein levels and the levels of IL-18 and IL-1β. A549 cells were treated with lipopolysaccharide (LPS) (10 μg/mL) for 24 h to simulate the inflammatory condition and SNX10 was knocked down using small interfering RNA. SNX10 knockdown cells showed increased viability and less ROS accumulation. Consistent with the *in vivo* results, the NF-κB/NLRP3 pathway and the secretion of inflammatory cytokines were inhibited after SNX10 knockdown in A549 cells. In summary, SNX10 downregulation mitigated sepsis-induced oxidative stress and pulmonary inflammation by inhibiting the NF-κB/NLRP3 pathway.

## Introduction

Sepsis is a common infectious condition characterized by concurrent organ failure (1). Its prevalence has been on the rise, and it continues to rank among the leading causes of death worldwide (2). Among the various organs affected, the lung is often the first and most frequently damaged (3), with acute lung injury (ALI) being a common complication in sepsis (4). ALI is marked by lung tissue dysfunction, extensive infiltration of inflammatory cells, progressive alveolar filling, and refractory arterial hypoxemia (5). Despite significant advances in understanding the pathophysiology of ALI over the past decade, the mortality rate remains high, and conventional treatment strategies have shown limited efficacy (6).

In the pathogenesis of sepsis-associated ALI, inflammation and oxidative stress play pivotal roles. Therefore, inhibiting the excessive production of inflammatory cytokines and reactive oxygen species (ROS) may hold promise for improving lung function and enhancing survival outcomes (7). During the development of ALI, the NLR family pyrin domain containing 3 (NLRP3) inflammasome is hyperactivated in macrophages (8), and prior studies have demonstrated that suppressing the NLRP3 inflammasome can alleviate sepsis-induced ALI (9). Additionally, the nuclear factor-κB (NF-κB) pathway stands out as one of the most classical and critical inflammatory signaling pathways in sepsis-induced ALI (10). Specifically, NF-κB signaling promotes the transcriptional activation of NLRP3 by upregulating the gene expression of NLRP3 inflammasome components (11). Once activated, the NLRP3 inflammasome facilitates the self-cleavage of pro-caspase-1 into its active form, thereby enhancing the maturation and release of the pro-inflammatory cytokines interleukin (IL)-1β and IL-18 (12).

Sorting nexin 10 (SNX10), a member of the sorting nexin family, contains a phox homology domain that interacts with phosphoinositides in membrane structures (13). It has been reported to be involved in various diseases, including cancer, inflammatory disorders, and metabolic diseases (14). Notably, SNX10 regulates the endosomal/lysosomal pathway, which is crucial for the inflammatory response (15). For example, studies have shown that SNX10 knockdown improves intestinal barrier dysfunction and inhibits inflammatory bowel disease (16), while SNX10 deficiency mitigates the progression of bone erosion in mice (17). Moreover, SNX10 knockdown is associated with reduced oxidative stress and inflammation, thereby alleviating alcohol-induced liver injury (18). In a mouse model, SNX10 has also been shown to modulate macrophage inflammatory responses and promote the development of colitis (15). Furthermore, SNX10 is highly expressed in mouse lung tissues, and its levels are further increased upon lipopolysaccharide (LPS) treatment (19). Emerging evidence emphasizes that lysosomal function and membrane integrity are pivotal for NLRP3 activation (20). For instance, the release of cathepsin B from damaged lysosomes is a potent activator of NLRP3 (21). Importantly, SNX10 knockdown has been proven to suppress the NF-κB signaling pathway and the activation of the NLRP3 inflammasome (22). These findings suggest that SNX10 may participate in the regulation of NLRP3 inflammasome function by modulating lysosomal membrane transport and stability.

In this study, we aimed to explore the function of SNX10 in mice with sepsis-induced ALI.

## Material and Methods

### Animals

All experiments were approved by the Laboratory Animal Welfare & Ethics Committee of Fujian Medical University (No. IACUC FJMU 2024-0254). All animal experiments complied with the ARRIVE guidelines. Eight-week-old male C57BL/6J mice (20-25 g) were obtained from Jiangsu Huachuang Sino Pharmaceutical Technology Co., Ltd., China. The mice were acclimatized for one week before use.

### Sepsis model

Sepsis was induced in mice via cecal ligation and puncture (CLP) to establish a model (23). After anesthesia, the abdominal skin was sterilized, the abdominal wall was incised along the midline, and the cecum was detached. The cecum was ligated at 75% using a 4-0 silk suture and punctured twice with a 1-mL syringe needle, avoiding areas with vascular density. Subsequently, the cecum was repositioned into the abdominal cavity. The abdominal incision was then closed with sutures. Sham-operated mice underwent the same laparotomy procedure, while the cecum was neither ligated nor perforated. Samples were collected 24 h after CLP induction.

### Cell treatments

Human lung epithelial cell line A549 cells were purchased from Shanghai Cellverse Cell Technology Co., Ltd., China. A549 cells were cultured with F-12K medium (Wuhan Servicebio Biotechnology Co., Ltd., China) containing 10% fetal bovine serum (Zhejiang Tianhang Biotechnology Co., Ltd., China). A549 cells were treated with 10 μg/mL LPS (Sigma-Aldrich Company, USA) for 24 h to simulate an inflammatory environment (24).

### SNX10 knockdown

C57BL/6J mice were anesthetized and the neck skin was disinfected. A minor incision was executed in the ventral cervical region of each mouse to expose the trachea. The adenovirus (1×10^8^ PFU) carrying short hairpin RNA (shRNA) plasmid targeting SNX10 (Hunan Fenghui Biotechnology Co., Ltd., China) was injected into the trachea in 50 μL phosphate buffer saline (PBS) (Biosharp, China) (25). CLP was conducted on mice after 72 h of infection.

The SNX10 small interfering RNA (siRNA) was obtained from Wuhan JTS Biotechnology Co., Ltd., China. The target sequence was as follows: AAUUCACAAGUUUGCCUUA (siSNX10). The siRNA fragments were transfected into A459 cells using Lipofectamine 3000 (Thermo Fisher Scientific Inc., USA). The cells were transfected for 24 h and then challenged with LPS for 24 h.

### Histological analysis

The fixed lung tissues were dehydrated with gradient alcohol, cleared with xylene (Shanghai Aladdin Biochemical Technology Co., Ltd., China), embedded with paraffin wax, sectioned into 5-μm slices, and stained with hematoxylin (Beijing Solarbio Technology Co., Ltd., China) and eosin (Shanghai Sangon Bioengineering Co., Ltd., China). According to Hirano et al. (26), we evaluated pulmonary injury by the histological score as follows: 0=none, 1=mild, 2=moderate, and 3=severe. Specific evaluation indicators were exudate, hyperemia/congestion, intra-alveolar hemorrhage/debris, cellular infiltration, and cellular hyperplasia. The pathological score was obtained by adding the scores of each index.

### Evaluation of lung edema

The wet weight was determined after cleaning the lung tissue surface using filter paper. Lung tissues were placed in an oven to dry at 65°C for 24 h and weighed again to determine the dry weight. The wet/dry weight ratio reflects the degree of pulmonary edema.

### Bronchoalveolar lavage fluid (BALF) total protein analysis

The skin of the mouse neck was cut and the trachea was separated. An oblique incision was made in the trachea, and 0.6 mL PBS was injected into the incision with a 1-mL syringe. The BALF was slowly aspirated for 30 s and this operation was repeated 3-5 times. We used the BCA method to evaluate pulmonary vascular permeability by measuring the protein concentration in BALF. After preparing the standard curve, appropriate samples were added to each well. Then, 200 μL of BCA working solution (Shanghai Beyotime Biotechnology Co., Ltd., China) was added to each well and incubated for 20 min at 37°C. A microplate reader (BioTek Instrument Co., Ltd., USA) was used to measure absorbance at a wavelength of 570 nm.

### Immunohistochemistry (IHC) analysis

Paraffin sections of lung tissue were dewaxed, subjected to antigen retrieval, incubated with hydrogen peroxide (Sinopharm Chemical Reagent Co., Ltd., China), and blocked with bovine serum albumin (BSA) (Shanghai Sangon Bioengineering Co., Ltd.). The sections were incubated with 1:100 SNX10 (Proteintech Group, Inc., USA) or 1:4000 F4/80 (Proteintech Group, Inc.) primary antibody at 4°C overnight, and 1:200 goat anti-rabbit IgG (Shanghai Sangon Bioengineering Co., Ltd.) at 25°C for 60 min. Following the diaminobenzidine color reaction (Beijing Solarbio Technology Co., Ltd.) and counterstaining with hematoxylin (Beijing Solarbio Technology Co., Ltd.), the stained samples were observed and photographed with an OLYMPUS microscope (Japan).

### p-p65^S536^ immunofluorescence (IF)

Lung tissues were sectioned into 5-μm paraffin sections. A549 cells were fixed with paraformaldehyde (Sinopharm Chemical Reagent Co., Ltd.) for 15 min and then treated with 0.1% tritonX-100 (Shanghai Beyotime Biotechnology Co., Ltd.) for 30 min. All samples were blocked with 1% BSA for 15 min, incubated with a 1:100 p-p65^S536^ primary antibody (ABclonal, Inc., China) at 4°C for 12 h, and with a 1:200 anti-rabbit IgG (Cell Signaling Technology, Inc., China) at 25°C for 60 min. The 4′,6-diamidino-2-phenylindole dihydrochloride (DAPI) (Shanghai Aladdin Biochemical Technology Co., Ltd.) staining was conducted for 5 min, followed by observation under a fluorescence microscope (OLYMPUS).

### Cell viability

A549 cells were plated onto 96-well plates at a seeding density of 4×10^3^ cells per well. MTT staining solution (50 μL) (Jiangsu Key GEN Biotechnology Co., Ltd., China) was added to each well and placed in the incubator. After 4 h, the supernatant was carefully aspirated, and 150 μL DMSO (Jiangsu Key GEN Biotechnology Co., Ltd.) was added to solubilize the purple crystals formed by the cells. Absorbance at 490 nm was measured using a microplate reader (BioTek Instrument Co., Ltd.).

### Cytokine measurement

All cytokine ELISA kits were purchased from Hangzhou Multi Sciences Biotechnology Co., Ltd., China. Lung tissues were homogenized in nine times their volume of saline. The cell supernatant and BALF were centrifuged at 300 *g* for 10 min at 4^o^C to remove sediment and were immediately analyzed. Three hundred μL of blood was obtained from the mouse orbital venous plexus, centrifuged at 862 *g* for 15 min at 4^o^C, and the supernatant was collected. The serum samples were analyzed immediately. The levels of IL-6, TNF-α, IL-18, and IL-1β in each sample were measured according to the manufacturer’s instructions.

### ROS detection

The lung tissues were cut into 10-μm-thick frozen slices and incubated with ROS probe reaction solution (Bestbio, China) at 37°C for 30 min. Slices were washed with PBS three times, and images were acquired using a fluorescence microscope (Olympus).

A549 cells were mixed with 1 mL of DCFH-DA diluent (Biotechnology Co., Ltd.), incubated at 37°C for 20 min with shaking every 5 min to mix. Cells were washed with PBS thrice, after which 500 μL of PBS was added. Cells were viewed with a fluorescence microscope (Olympus).

### Oxidative stress

The malondialdehyde (MDA), superoxide dismutase (SOD), and glutathione peroxidase (GSH) kits were obtained from Nanjing Jiancheng Bioengineering Institute (China). Lung tissues were mixed with 9 times their volume of normal saline, and mechanical homogenization was performed in an ice-water bath. The homogenates were then centrifuged at 383 *g* 10 min at 25°C, and oxidative stress levels were measured according to the manufacturer’s instructions.

### Real-time PCR

The SNX10 mRNA expression level was detected by real-time PCR. Total RNA was extracted from mouse lung tissues or A549 cells with TRIpure reagent (Beijing BioTeke Biotechnology Co., Ltd., China). The extracted total RNA was then reverse-trasncribed to cDNA using the All-in-One First-Strand SuperMix kit (Guangzhou Magen Biotechnology Co., Ltd., China). Real-time PCR was performed using a 2×Fast Taq plus PCR Master Mix (Biosharp, China) and 2×SYBR Green PCR Mastermix (Beijing Solarbio Technology Co., Ltd.). RNA expression levels were standardized to β-actin expression, and the data were analyzed using the 2^−ΔΔCt^ method. The SNX10 primer sequences were as follows: homo SNX10 F: AAGTAATGCGTTGCTGGTA; homo SNX10 R: AGGTGAAGGCTGCTATCT; mus SNX10 F: GCAGCCTCCACCTCTTCCT; mus SNX10 R: TCGGCGTCTTTCTTCCCT; homo β-actin F: TCAGGGTGAGGATGCCTCTC; homo β-actin R: CTCGTCGTCGACAACGGCT; mus β-actin F: GCCAGAGCAGTAATCTCCTTCT; mus β-actin R: AGTGTGACGTTGACATCCGTA.

### Immunoblotting

SNX10 antibody was purchased from Proteintech Group, Inc. p-p65, p65, NLRP3, and ASC antibodies were obtained from ABclonal, Inc. p20 caspase 1 and β-actin antibody were from Jiangsu Affinity Biosciences Research Center Co., Ltd. (China) and Santa Cruz Biotechnology, Inc. (USA), respectively. Lung tissues and A549 cell protein extracts were prepared for SDS-PAGE and electrophoresed to polyvinylidene fluoride membranes (Shanghai Abcam Trading Co., Ltd., China). After blocking, the membranes were incubated with the primary antibodies as follows: SNX10 (1:5000), p-p65 (1:5000), p65 (1:10000), NLRP3 (1:3000), ASC (1:3000), p20 caspase 1 (1:1000), and β-actin (1:1000) overnight at 4°C. Subsequently, the membranes were treated with 1:5000 anti-rabbit or anti-mouse secondary antibodies (Shanghai Beyotime Biotechnology Co., Ltd.) for 45 min at 37°C. The blots were subsequently identified using the ECL reagent (Shanghai Beyotime Biotechnology Co., Ltd.). The films were scanned, and the absorbance of the target band was analyzed using a GEL-Pro-Analyzer software.

### Analysis of the GEO database

The GSE15379 dataset was obtained from the GEO database (https://www.ncbi.nlm.nih.gov/geo/query/acc.cgi?acc=GSE15379), which contains expression data from the lungs of septic mice. In the GSE15379 dataset, lung samples from wild-type mice were collected at 8 h post-CLP (n=3) or sham surgery (n=3) for RNA extraction and Affymetrix microarray hybridization. Differentially expressed genes (DEGs) were identified using |log_2_ FC|>1 and P<0.05 as the screening criteria.

### Statistical analysis

All data are reported as means±SD. Graphs were generated using GraphPad Prism 8.5 (GraphPad Software, USA). Normality and log-normality tests were performed on all data. Discrepancies between two groups were analyzed by the unpaired *t*-test. Discrepancies among four groups were assessed using one-way ANOVA analysis followed by Tukey's multiple comparisons test. Welch's *t*-test or Brown-Forsythe and Welch ANOVA tests were used when the SDs were not equal. The Kruskal-Wallis test or the Mann-Whitney test was used for nonparametric data distribution. Statistical significance was defined as P<0.05.

## Results

### Sepsis-induced ALI upregulated SNX10 expression in the mouse lung tissues

**Figure 1:**
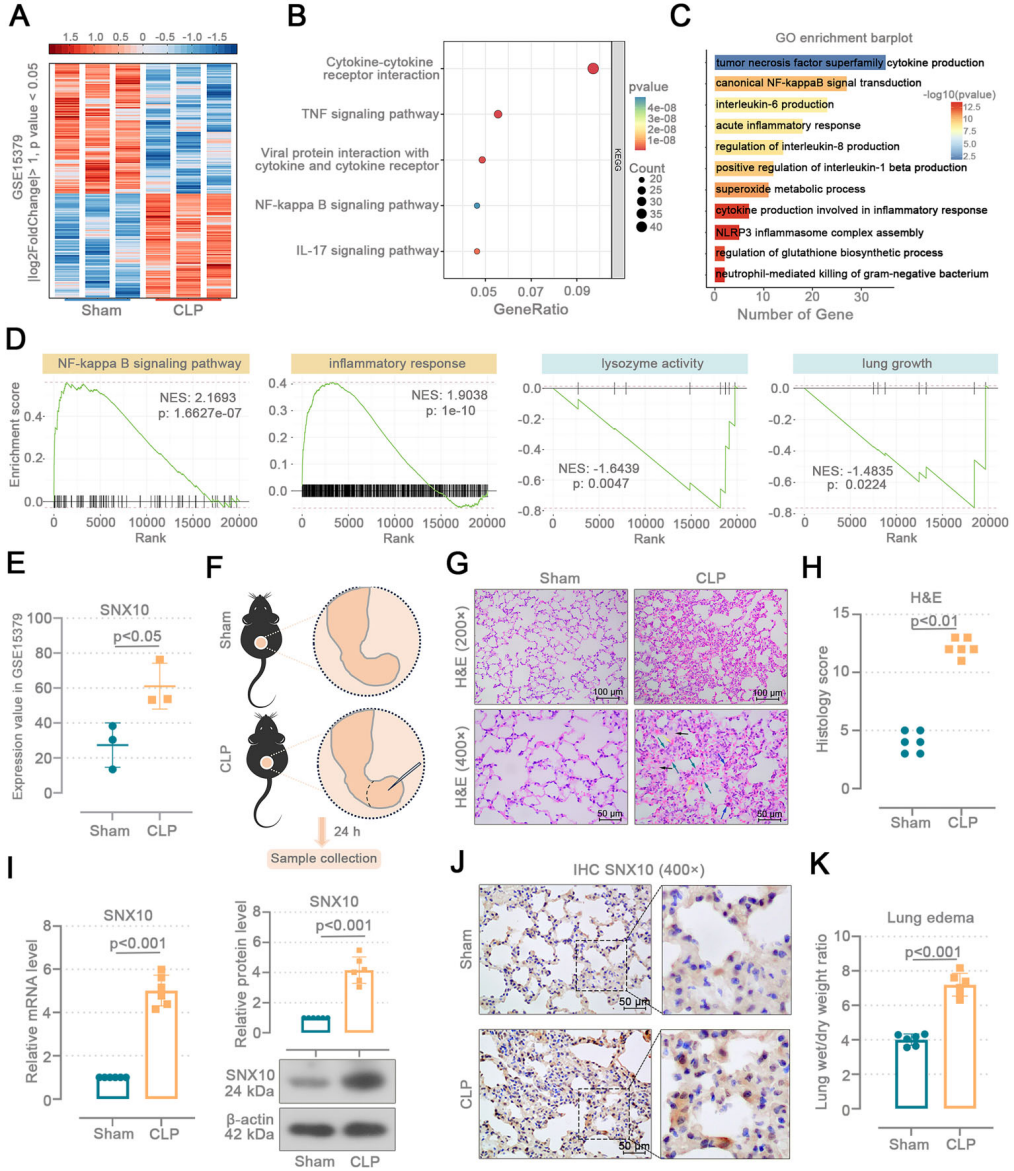
Sepsis-induced acute lung injury (ALI) upregulates the SNX10 expression in mouse lung tissues. In the GSE15379 database, lung samples were collected from cecal ligation and puncture (CLP) mice (n=3) or sham-operated mice (n=3) at 8-h post-procedure. **A**, Differentially expressed genes (DEGs) in the lung tissues of CLP mice and their control mice. **B**, Kyoto Encyclopedia of Genes and Genomes (KEGG) pathways enriched by the DEGs. **C**, Gene Ontology (GO) pathway enriched by the DEGs. **D**, Gene Set Enrichment Analysis (GSEA) of all genes. **E**, Expression values of SNX10 in the GSE15379 database. **F**, C57BL/6J mice were subjected to an abdominal sepsis model using cecal ligation and puncture (CLP). The cecum of sham mice was isolated without ligation or puncture. Lung tissues were collected 24 h after surgery. **G** and **H**, Histological analyses of H&E staining and lung injury score (Mann-Whitney test). The upper images were enlarged to 200× (scale bar=100 μm), and the lower images were enlarged to 400× (scale bar=50 μm). Yellow arrow: hemorrhage; black arrow: edema; blue arrow: inflammatory infiltration; green arrow: alveolar septal thickening. **I**, SNX10 mRNA and protein levels in the mouse lung tissues (Welch's *t*-test). **J**, Representative IHC images (400×) SNX10-stained mouse lung sections (scale bar=50 μm). **K**, Lung wet/dry weight ratio (unpaired *t*-test). Data are reported as means and SD.

We analyzed DEGs in the lung tissues from CLP and sham mice using the GSE15379 dataset (Figure 1A). Kyoto Encyclopedia of Genes and Genomes (KEGG) analysis revealed that DEGs were significantly enriched in pathways including cytokine-cytokine receptor interaction, TNF signaling pathway, and NF-κB signaling pathway (Figure 1B). Gene Ontology (GO) analysis of DEGs identified involvement in biological processes such as canonical NF-κB signal transduction, acute inflammatory response, and NLRP3 inflammasome complex assembly (Figure 1C). Gene Set Enrichment Analysis (GSEA) results demonstrated that the NF-κB signaling pathway and inflammatory response gene sets were upregulated, while lysozyme activity and lung growth gene sets were downregulated (Figure 1D). In the GSE15379 dataset, the expression level of SNX10 was elevated in the lung tissues of CLP mice (P<0.05) (Figure 1E).

A mouse model of sepsis-induced ALI was established via CLP (Figure 1F). We observed the pathological changes of the lung tissues by hematoxylin and eosin (H&E) staining and calculated the histology score (Figure 1G and H). CLP mice exhibited inflammatory infiltration, hemorrhage, thickening of alveolar septa, and edema of alveolar cells. The histological score of CLP mice was higher than that of sham mice (P<0.01). In mice subjected to CLP, SNX10 mRNA (P<0.01) and protein (P<0.001) levels were upregulated (Figure 1I). Representative IHC images show more sepia SNX10-positive cells in CLP mice than sham mice (Figure 1J). The pulmonary wet/dry weight ratio was increased (P<0.001), indicating pulmonary edema induced by CLP (Figure 1K). These results suggest that CLP-induced ALI promotes SNX10 expression in the mouse lung tissues.

### SNX10 knockdown alleviated lung damage in sepsis-induced ALI mice

**Figure 2:**
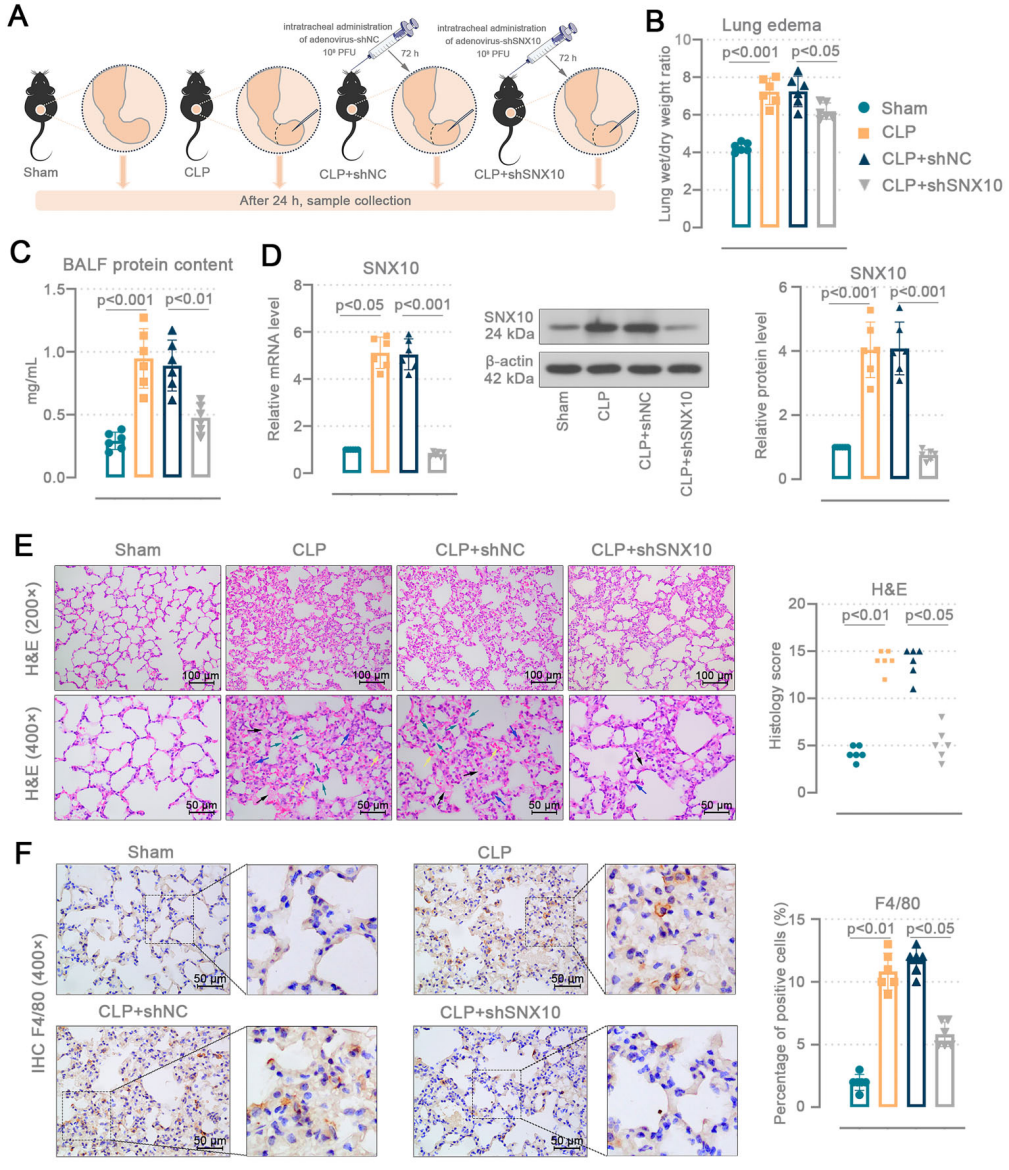
SNX10 knockdown alleviated lung damage in sepsis-induced acute lung injury (ALI) mice. **A**, C57BL/6J mice were injected intratracheally with the adenovirus containing SNX10 shRNA plasmids. After 72 h, the model of abdominal sepsis was induced by cecal ligation and puncture (CLP). The cecum of sham mice was isolated without ligation or puncture. The bronchoalveolar lavage fluid (BALF), lung tissues, and serum were collected 24 h after surgery. **B**, Lung wet/dry weight ratio. **C**, Protein content in the BALF. **D**, SNX10 mRNA (Kruskal-Wallis test and Dunn's multiple comparisons test) and protein (ordinary one-way ANOVA and Tukey's multiple comparisons test) levels in the mouse lung tissues. **E**, Histological analyses of H&E staining and lung injury score. Upper images were enlarged to 200× (scale bar=100 μm), and the lower images were enlarged to 400× (scale bar=50 μm). Yellow arrow: hemorrhage; black arrow: edema; blue arrow: inflammatory infiltration; green arrow: alveolar septal thickening. **F**, Representative IHC images of 400× F4/80-stained mouse lung sections and percentage of F4/80 positive cells (scale bar=50 μm). **B** and **C**: Ordinary one-way ANOVA and Tukey's multiple comparisons test; **E** and **F**: Data are reported as means and SD; Kruskal-Wallis test and Dunn's multiple comparisons test.

We conducted a knockdown of SNX10 in mice to explore its function in sepsis-induced ALI (Figure 2A). The severity of lung injury was evaluated by measuring pulmonary edema and the protein content in BALF. SNX10 knockdown reduced pulmonary edema in mice (P<0.05 *vs* shNC) (Figure 2B). CLP induced an elevation of BALF protein concentration (P<0.001 *vs* sham) in mice, suggesting serious damage to the pulmonary microvasculature, and this damage was attenuated (P<0.01 *vs* shNC) by SNX10 knockdown (Figure 2C). The knockdown efficiency of SNX10 is shown in Figure 2D and Supplementary Figure S1A-E. In CLP SNX10-deficient mice, pulmonary hemorrhage and inflammatory infiltration and alveolar cell edema were decreased and the histology score was lower than CLP mice (P<0.05 *vs* shNC) (Figure 2E). Figure 2F shows the F4/80 IHC stained results and percentage of F4/80-positive cells in the mouse lungs. CLP mouse lungs exhibited enhanced macrophage infiltration, indicated by an elevation in F4/80+ cells (P<0.01 *vs* sham). SNX10 knockdown decreased F4/80+ cells in the CLP mouse lung tissues (P<0.05 *vs* shNC). The findings reveal that SNX10 deficiency mitigates lung damage in sepsis-induced ALI mice.

### SNX10 knockdown mitigated lung inflammation and oxidative stress in sepsis-induced ALI mice

**Figure 3:**
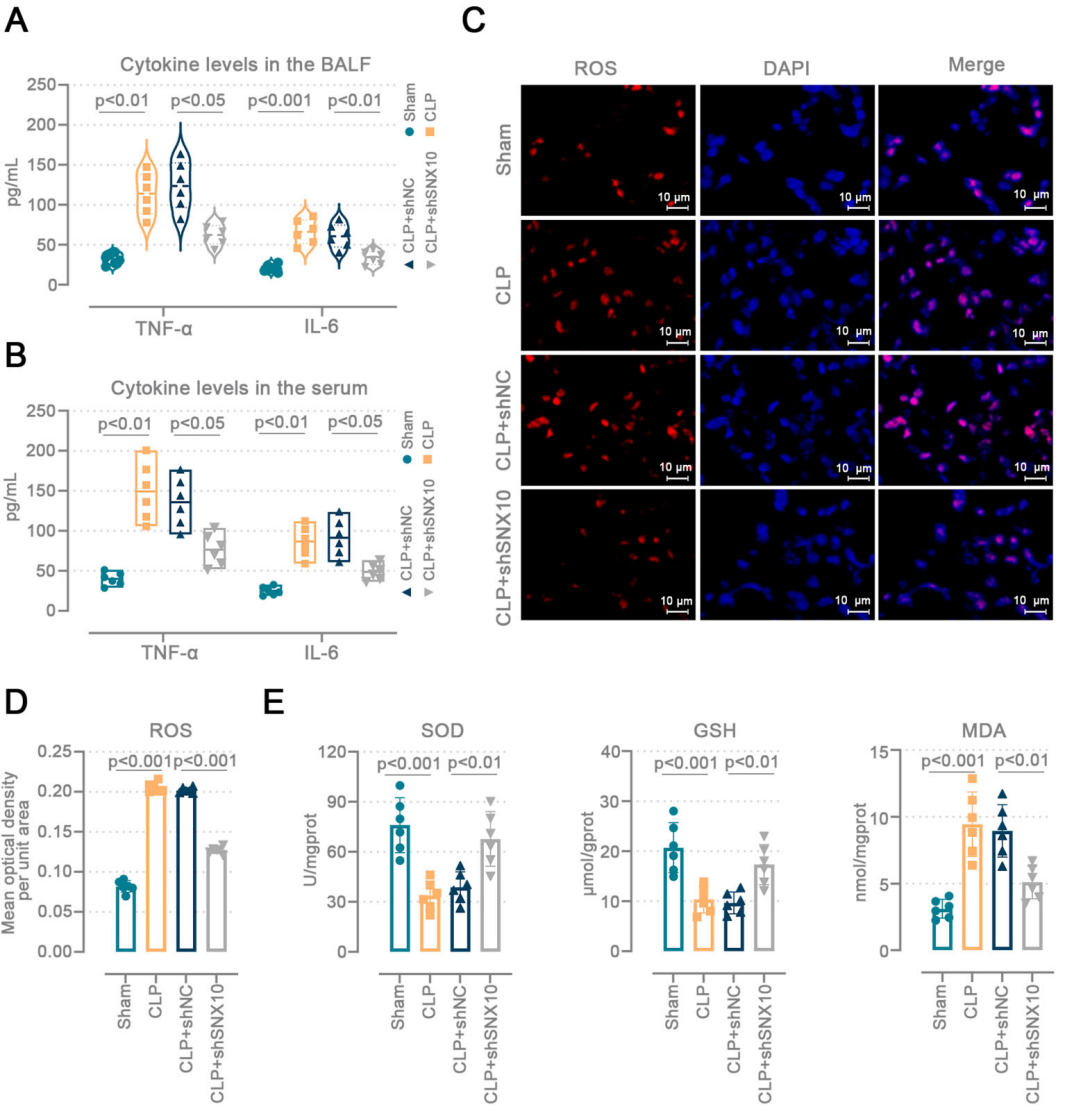
SNX10 knockdown mitigated lung inflammation and oxidative stress in sepsis-induced acute lung injury (ALI) mice. C57BL/6J mice were injected intratracheally with the adenovirus containing SNX10 shRNA plasmids. After 72 h, the abdominal sepsis model was induced by cecal ligation and puncture (CLP). The cecum of sham mice was isolated without ligation or puncture. Lung tissues and serum were collected 24 h after surgery. Tumor necrosis factor (TNF)-α (Brown-Forsythe and Welch ANOVA tests) and interleukin (IL)-6 (ordinary one-way ANOVA and Tukey's multiple comparisons test) levels in the (**A**) bronchoalveolar lavage fluid (BALF) and (**B**) in serum were determined. **C** and **D**, Representative images of 400× reactive oxygen species (ROS) staining and fluorescent quantitation in lung tissues (scale bar=10 μm). **E**, Oxidative stress levels in mouse lung tissues. SOD: superoxide dismutase; GSH: reduced glutathione; MDA: malondialdehyde. **D** and **E**: Ordinary one-way ANOVA and Tukey's multiple comparisons test. Data are reported as means and SD.

We assessed the degree of lung inflammation and oxidative stress by measuring cytokine levels, ROS levels, and antioxidant enzyme activity. CLP elevated TNF-α and IL-6 levels in the BALF and serum of mice, while SNX10 knockdown mitigated this alteration (Figure 3A and B). ROS-stained images showed that CLP mice had stronger lung ROS fluorescence intensity (P<0.001 *vs* sham), and the lack of SNX10 reduced ROS levels in the lung tissues (P<0.001 *vs* shNC) (Figure 3C and D). In CLP mice, SOD activity (P<0.001 *vs* sham) and GSH (P<0.001 *vs* sham) content declined while MDA (P<0.001 *vs* sham) content increased. SNX10 knockdown alleviated these effects (P<0.01) (Figure 3E). The results demonstrate that SNX10 deficiency reduced lung inflammation and oxidative stress in sepsis-induced ALI mice.

### SNX10 knockdown inhibited activation of the NF-κB/NLRP3 pathway in the lungs of sepsis-induced ALI mice

**Figure 4:**
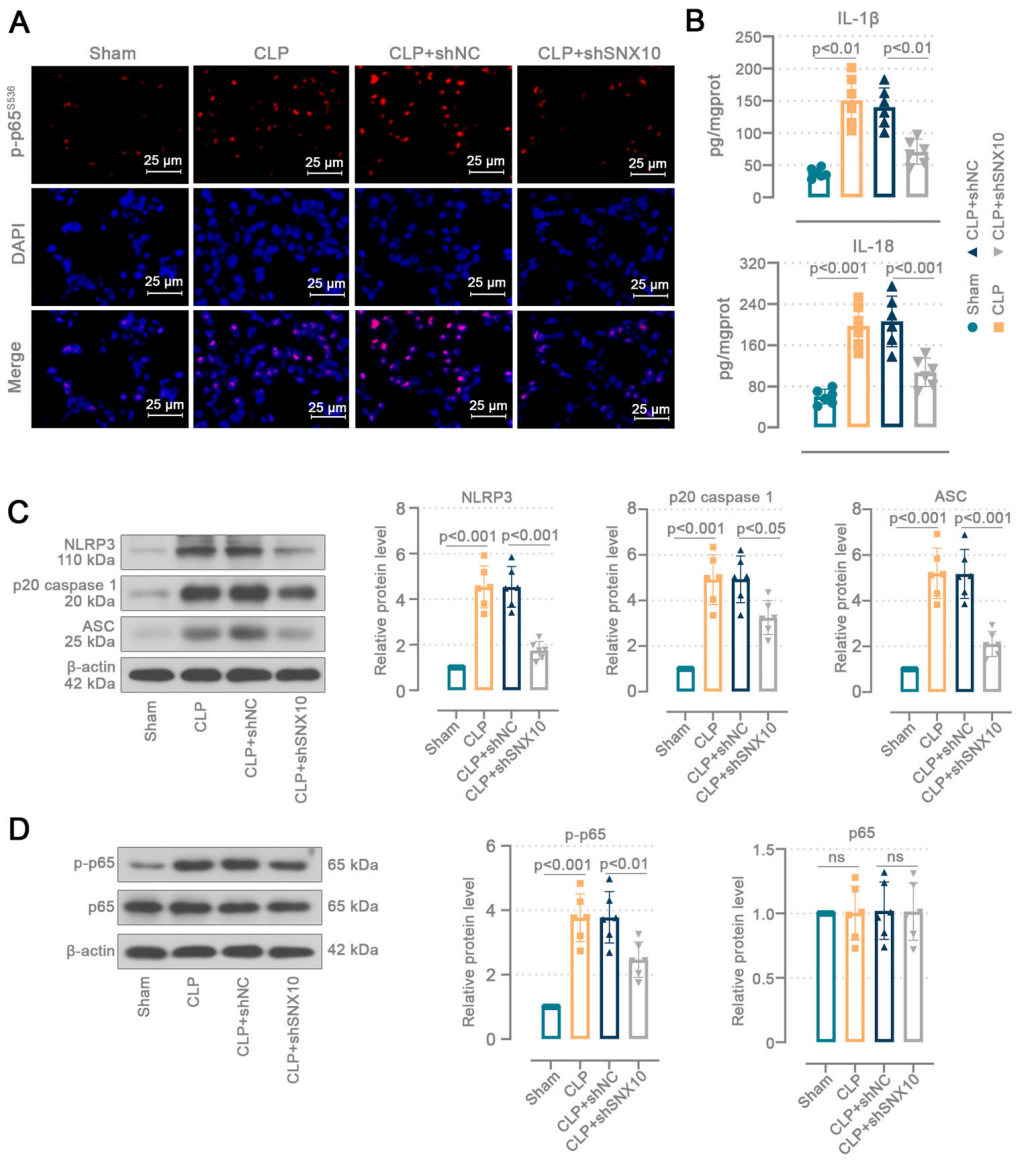
SNX10 knockdown inhibited activation of the NF-κB/NLRP3 pathway in the lungs of sepsis-induced acute lung injury (ALI) mice. C57BL/6J mice were injected intratracheally with the adenovirus containing SNX10 shRNA plasmids. After 72 h, the abdominal sepsis model was induced by cecal ligation and puncture (CLP). The cecum of sham mice was isolated without ligation or puncture. Lung tissues were collected 24 h after surgery. **A**, Immunofluorescence staining analysis of p-p65^s536^ nuclear localization in the mouse lung tissues (400×, scale bar=25 μm). **B**, Interleukin (IL)-1β (Brown-Forsythe and Welch ANOVA tests) and IL-18 (ordinary one-way ANOVA and Tukey's multiple comparisons test) levels in the mouse lung tissues. **C**, Protein levels of NLRP3, p20 caspase 1, and ASC (ordinary one-way ANOVA and Tukey's multiple comparisons test) in the mouse lung tissues. **D**, Protein levels of p65 (Kruskal-Wallis test and Dunn's multiple comparisons test) and p-p65 (ordinary one-way ANOVA and Tukey's multiple comparisons test) in the mouse lung tissues. Data are reported as means and SD. ns: not significant.

To further investigate the role of SNX10 in sepsis-induced ALI, we assessed relevant markers of the NF-κB/NLRP3 pathway. The red fluorescence intensity of p-p65^S536^ in CLP mice was more pronounced than in sham mice, and nuclear localization occurred for p-p65^S536^, whereas the expression of p-p65 declined in SNX10-deficient mice (Figure 4A). CLP mice displayed greater levels of IL-1β (P<0.01 *vs* sham) and IL-18 (P<0.001 *vs* sham) in pulmonary tissues (Figure 4B). After SNX10 knockdown, IL-1β (P<0.01 *vs* shNC) and IL-18 (P<0.001 *vs* shNC) levels were reduced in the mouse lungs. SNX10 knockdown attenuated CLP-induced elevations in NLRP3 (P<0.001 *vs* shNC), p20 caspase 1 (P<0.05 *vs* shNC), and ASC (P<0.001 *vs* shNC) protein levels in the pulmonary tissues (Figure 4C). CLP increased phosphorylated p65 protein levels in mice (P<0.001 *vs* sham) (Figure 4D). SNX10 downregulation decreased p65 phosphorylation in the CLP mouse lungs (P<0.01 *vs* shNC). These data prove that the SNX10 deficiency suppressed activation of the NF-κB/NLRP3 pathway in the lungs of sepsis-induced ALI mice.

### SNX10 knockdown ameliorated the damage to A549 cells caused by LPS

**Figure 5:**
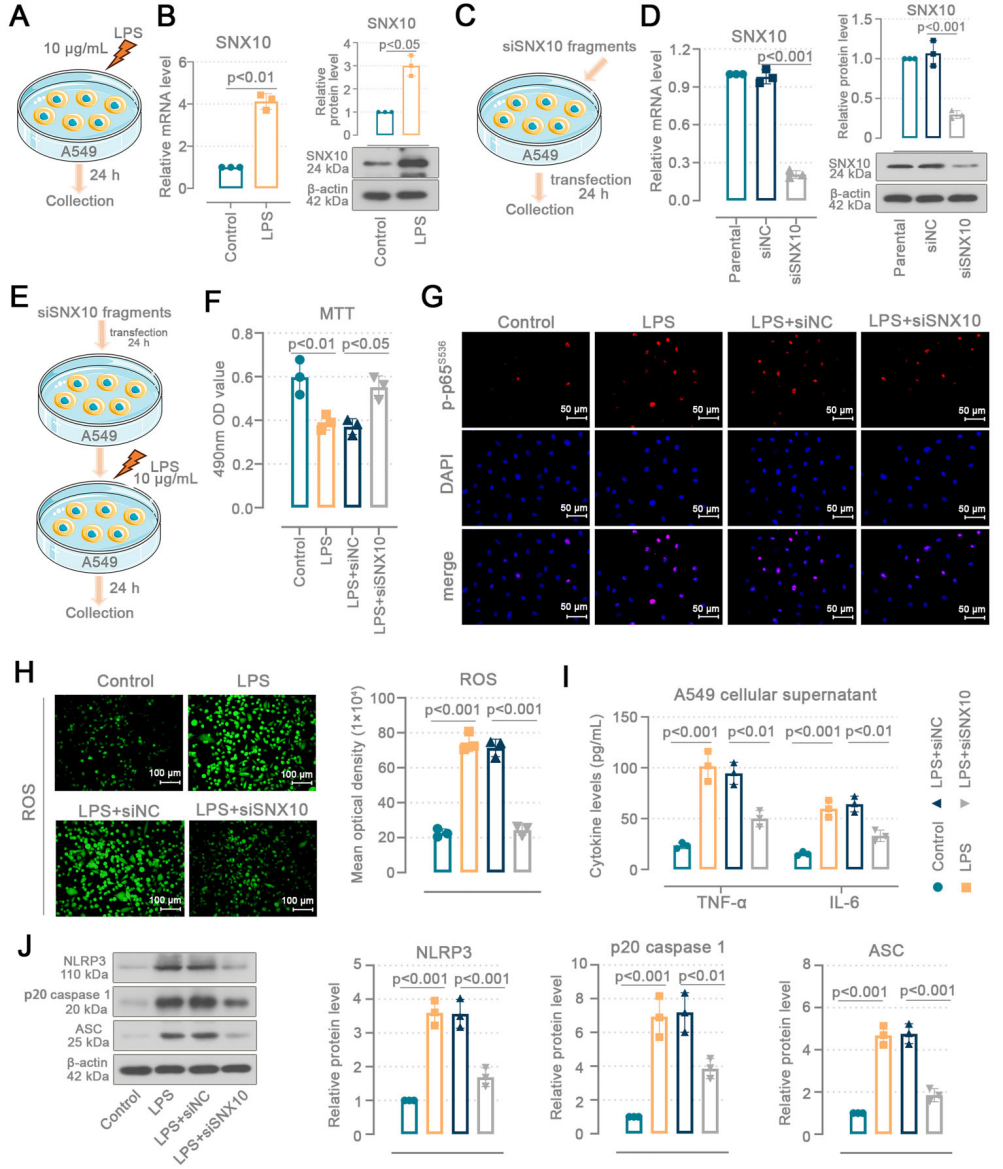
SNX10 knockdown ameliorated the damage to A549 cells caused by lipopolysaccharide (LPS). **A**, A549 cells were treated with LPS for 24 h. **B**, SNX10 mRNA and protein levels in A549 cells treated with LPS. **C**, A549 cells were transfected with siSNX10 and collected 24 h later. **D**, SNX10 mRNA and protein levels in A549 cells with SNX10 knockdown. **E**, A549 cells were transfected with siSNX10 and, after 24 h, treated with LPS for 24 h. **F**, MTT assay was conducted to measure cell viability. **G**, Immunofluorescence staining analysis of p-p65^s536^ nuclear localization in A549 cells (400×, scale bar=50 μm). **H**, Representative images of fluorescence probes (DCFH-DA) (200×, scale bar=100 μm) and quantification of reactive oxygen species (ROS) fluorescence intensity. **I**. Tumor necrosis factor (TNF)-α and interleukin (IL)-6 levels in A549 cellular supernatant. **J**, Protein levels of NLRP3, p20 caspase 1, and ASC in A549 cells. **B**: Welch's *t* test; **D**-**J**: ordinary one-way ANOVA and Tukey's multiple comparisons test. Data are reported as means and SD.

A549 cells were treated with LPS (Figure 5A) and SNX10 knockdown (Figure 5C) to investigate SNX10's role *in vitro*. SNX10 mRNA (P<0.01) and protein (P<0.05) levels were increased following LPS-challenged A549 cells (Figure 5B). The SNX10 knockdown efficiency (P<0.001) in A549 cells is shown in Figure 5D and Supplementary Figure S1F. A549 cells were transfected with the SNX10-targeted fragments followed by LPS treatment for 24 h (Figure 5E). LPS decreased the viability of A549 cells (P<0.01 *vs* control), and the cell viability was restored after SNX10 knockdown (P<0.05 *vs* siNC) (Figure 5F). More phosphorylated p65-positive cells were detected in cells treated with LPS and nuclear localization occurred for p-p65^S536^ (Figure 5G). SNX10 knockdown reduced p65 phosphorylation in A549 cells. SNX10 downregulation inhibited LPS-induced ROS accumulation in A549 cells (P<0.001 *vs* siNC) (Figure 5H). Levels of TNF-α and IL-6 were increased (P<0.001 *vs* control) in the supernatant of LPS-treated cells and this increase was ameliorated (P<0.01 *vs* siNC) following SNX10 knockdown (Figure 5I). Consistent with *in vivo* results, LPS-induced increases in NLRP3 (P<0.001 *vs* sham), p20 caspase 1 (P<0.001 *vs* sham), and ASC (P<0.001 *vs* sham) protein levels were reversed (P<0.001, P<0.01, P<0.001 *vs* siNC, respectively) after SNX10 knockdown in A549 cells (Figure 5J). These results confirmed that SNX10 knockdown reduced LPS-induced damage to A549 cells.

## Discussion

Our novel data indicated that SNX10 knockdown alleviated lung inflammation and oxidative stress. The specific mechanism is that SNX10 absence inhibits the activation of the NF-κB/NLRP3 pathway and decreases ROS levels. These findings suggest that SNX10 may represent a key target for alleviating sepsis-induced ALI.

In sepsis-induced ALI, the dysfunctional alveolar cells release inflammatory mediators and recruit leukocytes (10). Leukocytes, particularly neutrophils and monocytes, infiltrating the lung parenchyma magnify the preexisting imbalance between pro-inflammatory and anti-inflammatory responses due to an overactive immune response. Cytokine storm is produced, causing damage to blood vessels and lungs (27). An excessive inflammatory response leads to heightened pulmonary vascular permeability and elevated lung interstitial protein levels, culminating in pulmonary edema (28).

In this study, we established a sepsis-induced ALI mouse model using the CLP method. Consistent with a previous report (29), CLP mice exhibited pulmonary hemorrhage, inflammatory infiltration, edema, thickening of the alveolar septum, and elevated protein levels in BALF. SNX10 expression is significantly increased in lung cancer patients with an unfavorable prognosis (30). According to Lou et al.'s study (19), SNX10 was expressed at elevated levels in murine lung macrophages and was upregulated when bacterial infection occurred. CLP-induced ALI mice exhibited increased SNX10 mRNA and protein levels, suggesting that SNX10 was involved in sepsis pathophysiology. We infected mice with the adenovirus containing the shSNX10 plasmid. SNX10 knockdown reduced BALF protein content and pulmonary edema in CLP mice. The number of macrophages decreased, and inflammation was relieved in CLP mice with SNX10 knockdown. The results suggest that SNX10 deficiency mitigates the effects of CLP on the mouse lungs.

The NLRP3 inflammasome is a multiprotein complex involved in the immune response and the interaction of ASC with NLRP3 leads to the formation of an ASC dimer, resulting in a speck-like structure. In addition, ASC interacts with caspase 1 to oligomerize it, transforming the inactive precursor enzyme into an active form of caspase 1 (31). The active form of caspase 1 facilitates the release of IL-1β and IL-18. The inflammatory cytokines IL-1β and IL-18 promote ALI's pathogenesis thorough inflammasome activation products (32). CLP triggered the activation of the NLRP3 inflammasome in mice and promoted the secretion of IL-1β and IL-18 (33). Our results showed that NLRP3, ASC, and p20 caspase 1 protein levels were increased in the lungs of mice challenged with CLP, accompanied by increased levels of IL-1β and IL-18. Sepsis-induced ALI was improved by inhibiting NLRP3 inflammasome activation (34). SNX10 knockdown inhibited NLRP3 activation and mitigated the release of IL-1β and IL-18 in CLP mouse lungs. Inhibition of SNX10 also exhibited the same anti-NLRP3 activation properties in LPS-induced A549 cells.

Bacterial stimulation induces the release of NF-κB dimers and the nuclear translocation of p65, which is a crucial element of NF-κB signaling (35). NF-κB can bind to specific DNA sequences and activate the transcription of many genes that will enhance the levels of pro-inflammatory cytokines, including TNF-α and IL-6 (36). In addition, NF-κB regulates the activation of the NLRP3 inflammasome and the synthesis of IL-1β (31). The persistent activation of NF-κB correlates with the degree of lung injury, and the suppression of NF-κB signaling is beneficial for efficiently mitigating lung inflammation (37). Consistent with previous findings (38), CLP induced the expression of lung NF-κB p-p65 protein and increased levels of TNF-α and IL-6 in mouse serum, BALF, and A549 cell supernatant. The NF-κB pathway was inhibited and TNF-α and IL-6 were reduced in SNX10-deficient mouse lung epithelial cells.

Excessive generation of ROS impairs pulmonary epithelial function and leads to epithelial cell death (39). Sepsis-induced ROS are vital to stimulate the NF-κB/NLRP3 pathway (35). We detected elevated ROS levels, decreased GSH and SOD activity, and increased MDA content in CLP mouse lungs. SNX10 knockdown increased antioxidant enzyme activity, restored ROS levels, and raised viability in mouse lung epithelial cells. According to these findings, SNX10 deficiency restrains oxidative stress in alveolar epithelial cells, which in turn prevents the NF-κB/NLRP3 pathway from being activated.

Many broad-spectrum anti-inflammatory therapies failed in sepsis trials, often due to excessive immunosuppression that impairs host defense (40). By targeting an upstream regulator of membrane trafficking rather than the inflammasome or cytokines directly, we aim to achieve more precise modulation: specifically dampening overactive NLRP3 signaling by SNX10 knockdown while preserving broader immune competence. This strategy could avoid the pitfalls of global immunosuppression, offering a more balanced approach to mitigating ALI in sepsis. However, there are some limitations in our work. For adenovirus-mediated delivery, SNX10 expression was modulated via intratracheal injection. This delivery route ensured preferential targeting of the viral vector to the lung and reduced its widespread dissemination to distant organs through systemic circulation. Nonetheless, the possibility of off-target effects on SNX10 expression in other tissues cannot be entirely ruled out. Additionally, since the health of the lung is dependent on the interaction between epithelial cells, vascular endothelial cells, and immune cells, the protective effects of SNX10 absence need to be explored in other cells as well.

In conclusion, our study revealed that the absence of SNX10 had a mitigation effect on ALI. SNX10 knockdown reduced oxidative stress levels and alleviated inflammation by inhibiting the activation of the NF-κB/NLRP3 pathway in the alveolar epithelial cells. The current results provide a possible new target for the treatment of ALI.

## Supplementary Materials

Supplementary Material Click here to view [pdf].

## Data Availability

All raw data generated in this study are available upon request from the corresponding authors.
