# Supplementary material for: Sorting nexin 10 knockdown: new strategies for alleviating sepsis-associated acute lung injury
Source: Braz J Med Biol Res. 2026 Feb 16;59:e15117. doi: 10.1590/1414-431X2025e15117 (PMC12919756; doi:10.1590/1414-431X2025e15117)

Figure S1. Validation of SNX10 knockdown. C57BL/6J mice were injected intratracheally with the adenovirus containing SNX10 shRNA plasmids. After 72 h, the model of abdominal sepsis was induced by cecal ligation and puncture (CLP). **A**–**E**, SNX10 protein levels in the mouse lung tissues. **F**, A549 cells were transfected with siSNX10 fragments and were subsequently collected 24 h later. SNX10 protein levels in A549 cells.

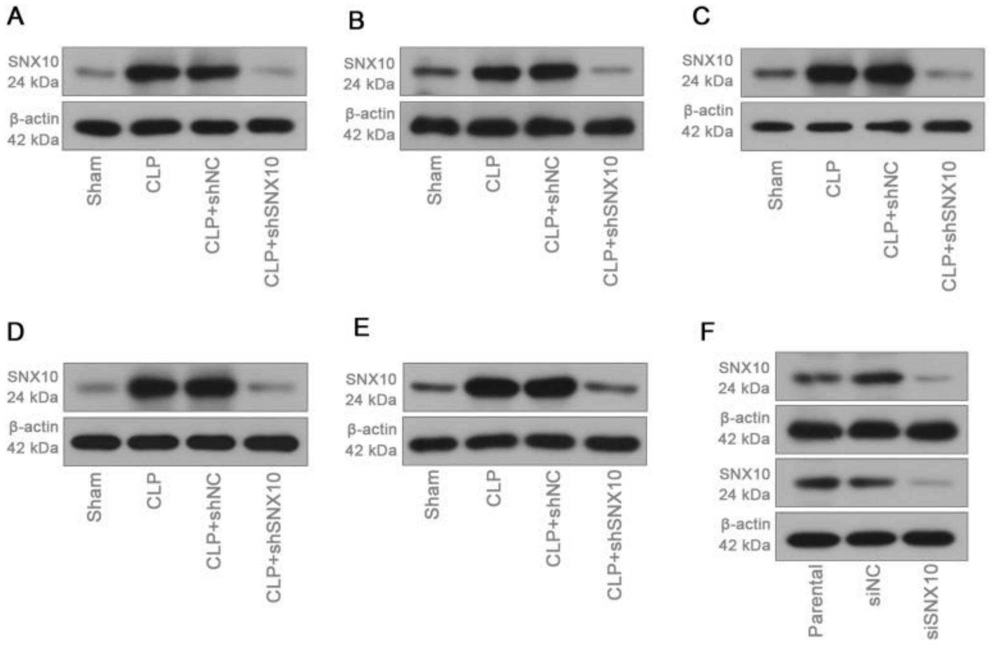

Supplement: Supplementary Material [file 1414-431X-bjmbr-59-e15117-suppl.pdf]
